# Identification and Functional Analysis of Long Non-coding RNAs in Human Pulmonary Microvascular Endothelial Cells Subjected to Cyclic Stretch

**DOI:** 10.3389/fphys.2021.655971

**Published:** 2021-04-01

**Authors:** Dong Wang, Chenyang Dai, Xiaoning Zhang, Changping Gu, Mengjie Liu, Huan Liu, Fan Yang, Haifeng Wu, Yuelan Wang

**Affiliations:** ^1^Department of Anesthesiology, The First Affiliated Hospital of Shandong First Medical University & Shandong Provincial Qianfoshan Hospital, Shandong Institute of Anesthesia and Respiratory Critical Medicine, Jinan, China; ^2^Department of Ophthalmology, Qilu Hospital of Shandong University, Jinan, China; ^3^Department of Anesthesiology, Shandong Provincial Qianfoshan Hospital, Shandong University, Cheeloo College of Medicine, Jinan, China

**Keywords:** lncRNA, expression profile, cyclic stretch, human pulmonary microvascular endothelial cell, *MMP1*, *TNFAIP3*, *TLR4*

## Abstract

**Background:** Despite decades of intense research, the pathophysiology and pathogenesis of acute respiratory distress syndrome (ARDS) are not adequately elucidated, which hamper the improvement of effective and convincing therapies for ARDS patients. Mechanical ventilation remains to be one of the primary supportive approaches for managing ARDS cases. Nevertheless, mechanical ventilation leads to the induction of further aggravating lung injury which is known as leading to ventilator-induced lung injury (VILI). It has been reported that lncRNAs play important roles in various cellular process through transcriptional, posttranscriptional, translational, and epigenetic regulations. However, to our knowledge, there is no investigation of the expression profile and functions of transcriptome-level endothelium-related lncRNAs in VILI yet.

**Methods:** To screen the differential expression of lncRNAs and mRNAs in Human pulmonary microvascular endothelial cells (HPMECs) subjected to cyclic stretch, we constructed a cellular model of VILI, followed by transcriptome profiling using Affymetrix *Human Transcriptome Array 2.0*. Bioinformatics analyses, including functional and pathway enrichment analysis, protein–protein interaction network, lncRNA-mRNA coexpression network, and cis-analyses, were performed to reveal the potential functions and underlying mechanisms of differentially expressed lncRNAs.

**Results:** In total, 199 differentially expressed lncRNAs (DELs) and 97 differential expressed mRNAs were screened in HPMECs subjected to 20% cyclic stretch for 2 h. The lncRNA-mRNA coexpression network suggested that DELs mainly enriched in response to hypoxia, response to oxidative stress, inflammatory response, cellular response to hypoxia, and NF-kappa B signaling pathway. LncRNA *n335470, n406639, n333984*, and *n337322* might regulate inflammation and fibrosis induced by cyclic stretch through cis- or trans-acting mechanisms.

**Conclusion:** This study provides the first transcriptomic landscape of differentially expressed lncRNAs in HPMECs subjected to cyclic stretch, which provides novel insights into the molecular mechanisms and potential directions for future basic and clinical research of VILI.

## Introduction

Acute respiratory distress syndrome (ARDS) is a life-threatening clinical syndrome characterized by non-cardiogenic pulmonary edema and severe hypoxemia. The common etiologies of ARDS are sepsis, pneumonia, and aspiration of gastric contents. Recent studies demonstrated a progressive decline of the incidence of ARDS, accounting for 10% in the intensive care unit globally. However, the mortality of ARDS was still as high as 30–40% in many countries (Bellani et al., 2016; Pham and Rubenfeld, 2017; Matthay et al., 2019). Despite decades of intense research, the pathophysiology and pathogenesis of ARDS are not adequately elucidated, which hamper the improvement of effective and convincing therapies for ARDS patients. Mechanical ventilation remains to be one of the primary supportive approaches for managing ARDS cases (Fan et al., 2018; Matthay et al., 2020). Nevertheless, mechanical ventilation leads to the induction of further aggravate lung injury which is known as leading to ventilator-induced lung injury (VILI) (Moraes et al., 2014; Bates and Smith, 2018). However, the underlying mechanisms of endothelial dysfunction in VILI are still unclear.

Deciphering transcriptional regulation are critical for understanding the molecular mechanisms of majority of biological processes. It has been suggested that lncRNAs, which comprised the majority of transcriptome, play important roles in various cellular process through transcriptional, posttranscriptional, translational, and epigenetic mechanisms (Rinn and Chang, 2012; Batista and Chang, 2013; Quinn and Chang, 2016; Tao et al., 2016). Recent studies have demonstrated that lncRNA *MALAT1, PRNCR1, THRIL*, and *CASC9*, acting as a competitive endogenous RNA, played key regulatory roles in sepsis-induced acute lung injury (Chen et al., 2020; Nan et al., 2020; Wang et al., 2020; Yu et al., 2020). It has been proposed that *NEAT1* and *lncRNA*-*5657* provided attractive targets for developing therapeutic strategy for ARDS (Liu et al., 2020; Zhou et al., 2020). To our knowledge, there is no investigation study of the expression profile and functions of transcriptome-level endothelium-related lncRNAs in VILI yet.

In the study, we used the Affymetrix Human Transcriptome Array 2.0 to screen differentially expressed lncRNAs and mRNAs in HPMECs subjected to cyclic stretch. Bioinformatics analyses, including functional and pathway enrichment analysis, protein–protein interaction network, cis-analyses of lncRNA, and lncRNA-mRNA coexpression network, were performed to explore the potential functions and underlying mechanisms of differentially expressed lncRNAs.

The findings provide new insights into the molecular regulatory mechanisms of lncRNAs in HPMECs exposed to cyclic stretch, and might contribute to the discovery of new therapeutic targets.

## Materials and Methods

### Cell Culture and Cyclic Stretch

Human pulmonary microvascular endothelial cells (HPMECs, ScienCell, San Diego, CA, USA) were cultured according to the manufacturer's instructions. Based on previous literature (Abiko et al., 2015; Meliton et al., 2015; Tian et al., 2016), HPMECs were subjected to 20% cyclic stretch for 0 or 2 h using the FX-5000T Flexercell Tension Plus system (Flexcell International, McKeesport, PA) (*n* = 3 per group).

### RNA Extraction and Microarray Hybridization

HPMECs were harvested for RNA extraction using Trizol reagent (Thermo Fisher Scientific, Waltham, MA, United States). RNA quantification and purity were determined using a Nanodrop ND-2000. Then, cDNA labeling and microarray hybridization were performed by the Genminix Informatics Company (Shanghai, China).

### Differential Expression Analysis

GCBI (https://www.gcbi.com.cn), which is a comprehensive bioinformatics analysis platform, provides data analysis online based on R programming language. The microarray data were pre-processed using the Robust Multi-array Average (RMA) method for background-corrected, normalization, and summary on the GCBI bioinformatics platform (Kong et al., 2016; Huang et al., 2018). Subsequently, Significant Analysis of Microarray (SAM) algorithm was used to identify differentially expressed lncRNAs (DELs) and differentially expressed genes (DEGs) with the fold change cutoff of 1.2 and *p*-value cutoff of 0.05 according to the prior reported literatures (Jia and Zhai, 2019; Li et al., 2020; Zhang et al., 2020). Hierarchical clustering was performed to distinguish the different gene clustering patterns on the same platform.

### Functional and Pathway Enrichment Analysis

Kyoto Encyclopedia of Genes and Genomes (KEGG) pathway and Gene Ontology (GO) functional enrichment analysis were performed using The Database for Annotation, Visualization, and Integrated Discovery (DAVID v6.8, https://david.ncifcrf.gov) (Huang da et al., 2009a,b; Jiao et al., 2012). GO includes three items: cellular component, molecular function, and biological process. *P* < 0.05 was considered to be statistically significant.

### Protein–Protein Interaction Network Construction and Module Analysis

PPI network was constructed in the Search Tool for the Retrieval of Interacting Genes/ Proteins database (STRING v11.0, https://string-db.org) with minimum required interaction score set in 0.4 (Szklarczyk et al., 2019), and visualized in the Cytoscape (V3.8.2) subsequently (Shannon et al., 2003; Otasek et al., 2019). Module analysis of PPI network was conducted by the MCODE application in Cytoscape, with node score cut off >3.0 (Bader and Hogue, 2003).

### LncRNA-mRNA Coexpression Network

LncRNA-mRNA coexpression network was constructed in the GCBI online analysis tool and visualized in the Cytoscape (V3.8.2), subsequently. The functional enrichment analysis of the DEGs in the lncRNA-mRNA coexpression network was performed by the DAVID online tool with a significance threshold of *p* < 0.05.

### Cis-Analyses of Differentially Expressed lncRNAs

UCSC Genome Bioinformatics tool (http://genome.ucsc.edu) was used to classify lncRNA cis-target genes. We searched genes within 10k upstream and downstream of DELs, and found the adjacent mRNAs and then analyzed their differential expression.

## Results

### Identification of Differentially Expressed lncRNAs and mRNAs

**Figure 1:**
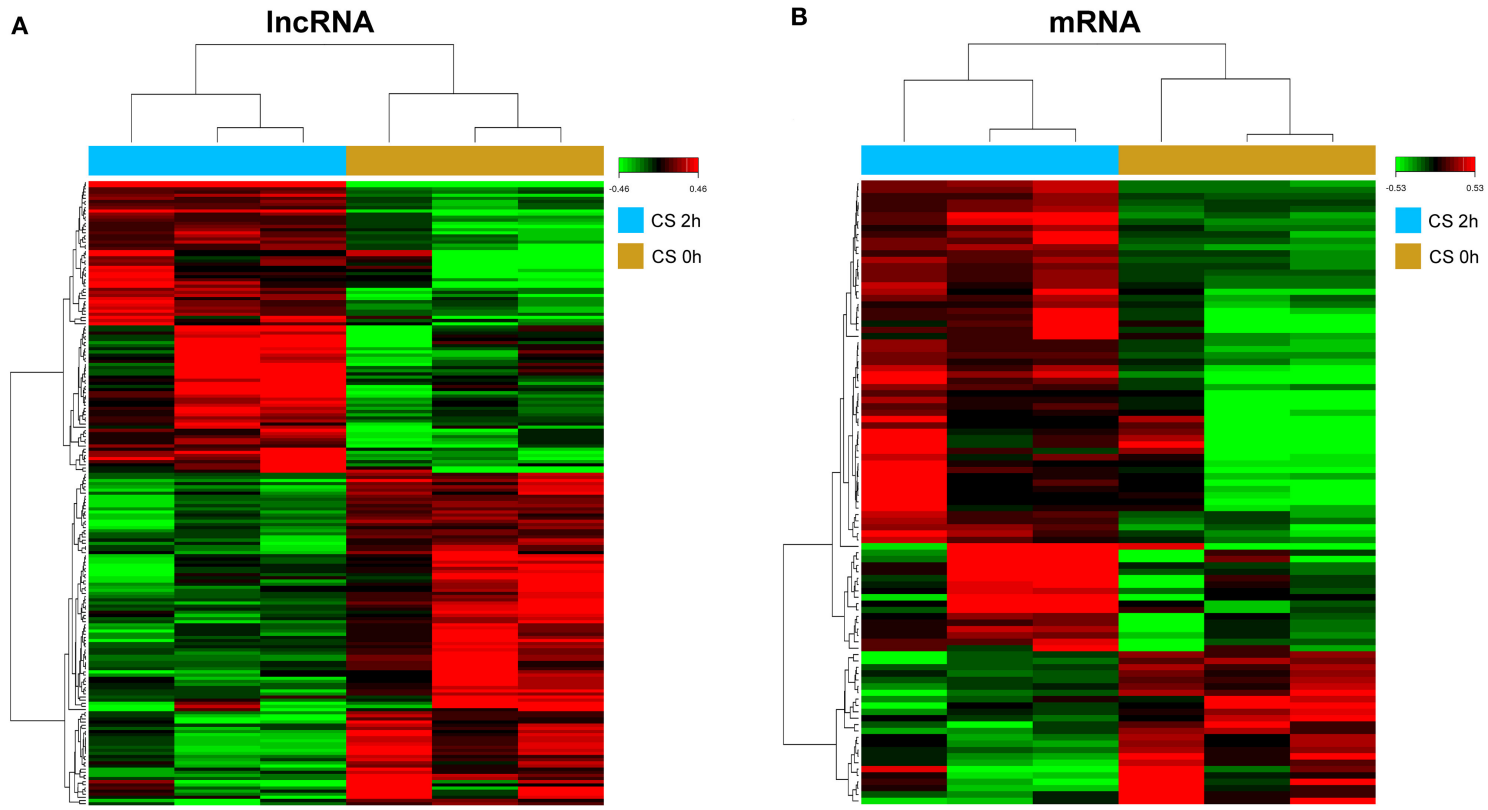
Hierarchical clustering of differentially expressed lncRNAs (DELs) **(A)** and differentially expressed genes (DEGs) **(B)**. Red to green colors refer to be high to low relative expression levels.

To screen the differential expression of mRNAs and lncRNAs in HPMECs subjected to cyclic stretch, we performed high-throughput analysis using the Affymetrix *Human Transcriptome Array 2.0*. After data preprocessing, a total of 199 lncRNAs were determined to be differentially expressed (Figure 1A; Supplementary Material 1). Among these lncRNAs, 93 lncRNAs were upregulated and 106 lncRNAs were downregulated. At the same time, 97 mRNAs were significantly differentially expressed (Figure 1B; Supplementary Material 2), including 74 upregulated mRNAs and 23 downregulated mRNAs.

### GO Functional and KEGG Pathway Enrichment Analysis

**Figure 2:**
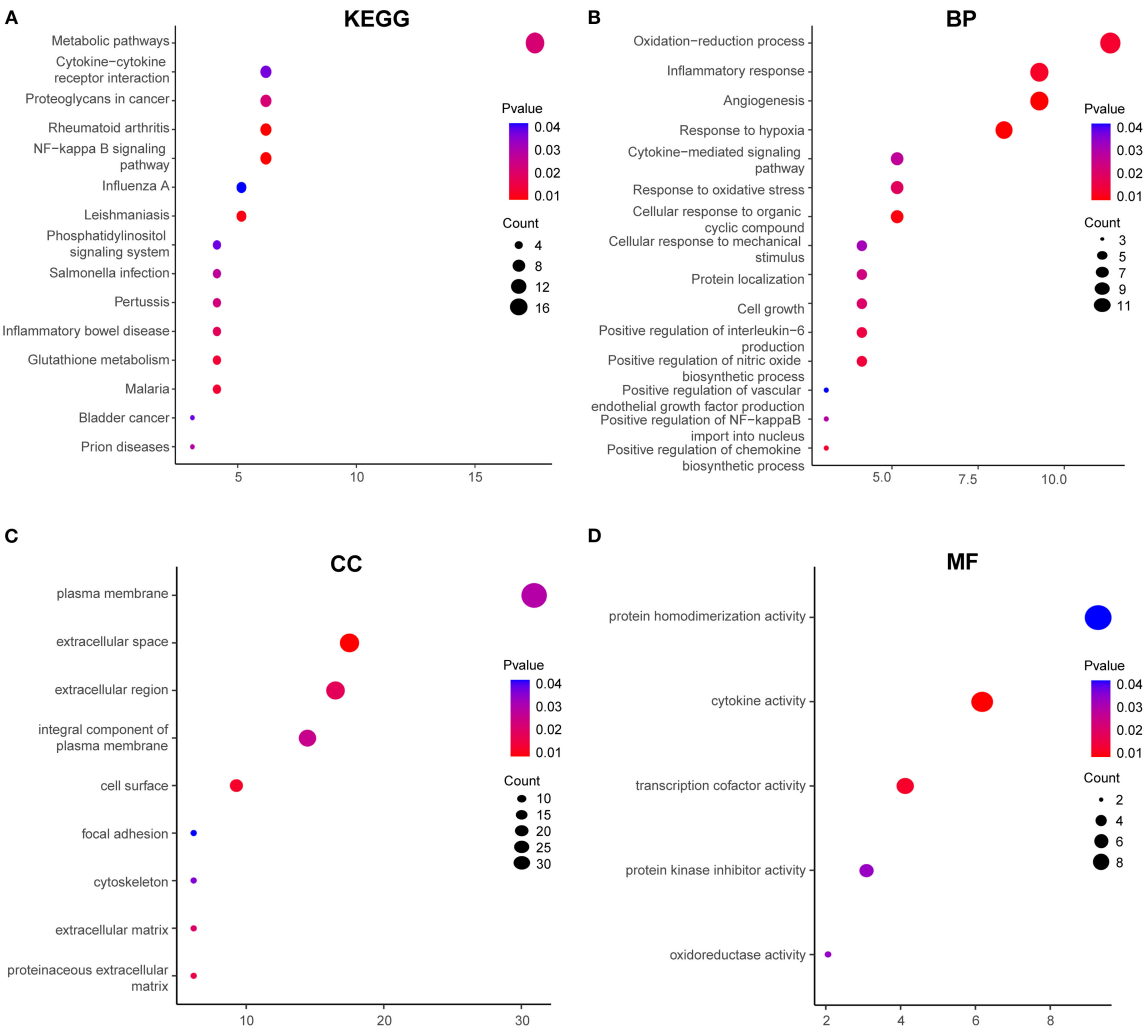
GO functional and KEGG enrichment analyses of 97 DEGs. **(A)** Significantly enriched KEGG pathways. GO functional enrichment analysis includes biological process (BP) **(B)**, cellular component (CC) **(C)**, and molecular function (MF) **(D)**. Red to blue colors indicate low to high levels of *p*-value. Point size indicates the number of DEGs in the corresponding pathway.

To explore the biological processes and pathways of DEGs in HPMECs exposed to cyclic stretch, GO functional and KEGG pathway enrichment analyses were performed. KEGG pathway enrichment analysis revealed that the NF-kappa B signaling pathway was the most significantly enriched pathway (Figure 2A). GO functional enrichment analysis showed that DEGs were mainly enriched in biological processes (BP) of oxidation-reduction process, inflammatory response, angiogenesis, response to hypoxia, cytokine-mediated signaling pathway, response to oxidative stress, and cellular response to mechanical stimulus (Figure 2B). As for cell component (CC), DEGs showed enrichment in plasma membrane, extracellular space, extracellular region, integral component of plasma membrane, and cell surface (Figure 2C). Besides, molecular function (MF) analysis indicated enrichment predominantly at cytokine activity and transcription cofactor activity (Figure 2D).

### PPI Network Construction, Module Analysis, and Hub Gene Selection

**Figure 3:**
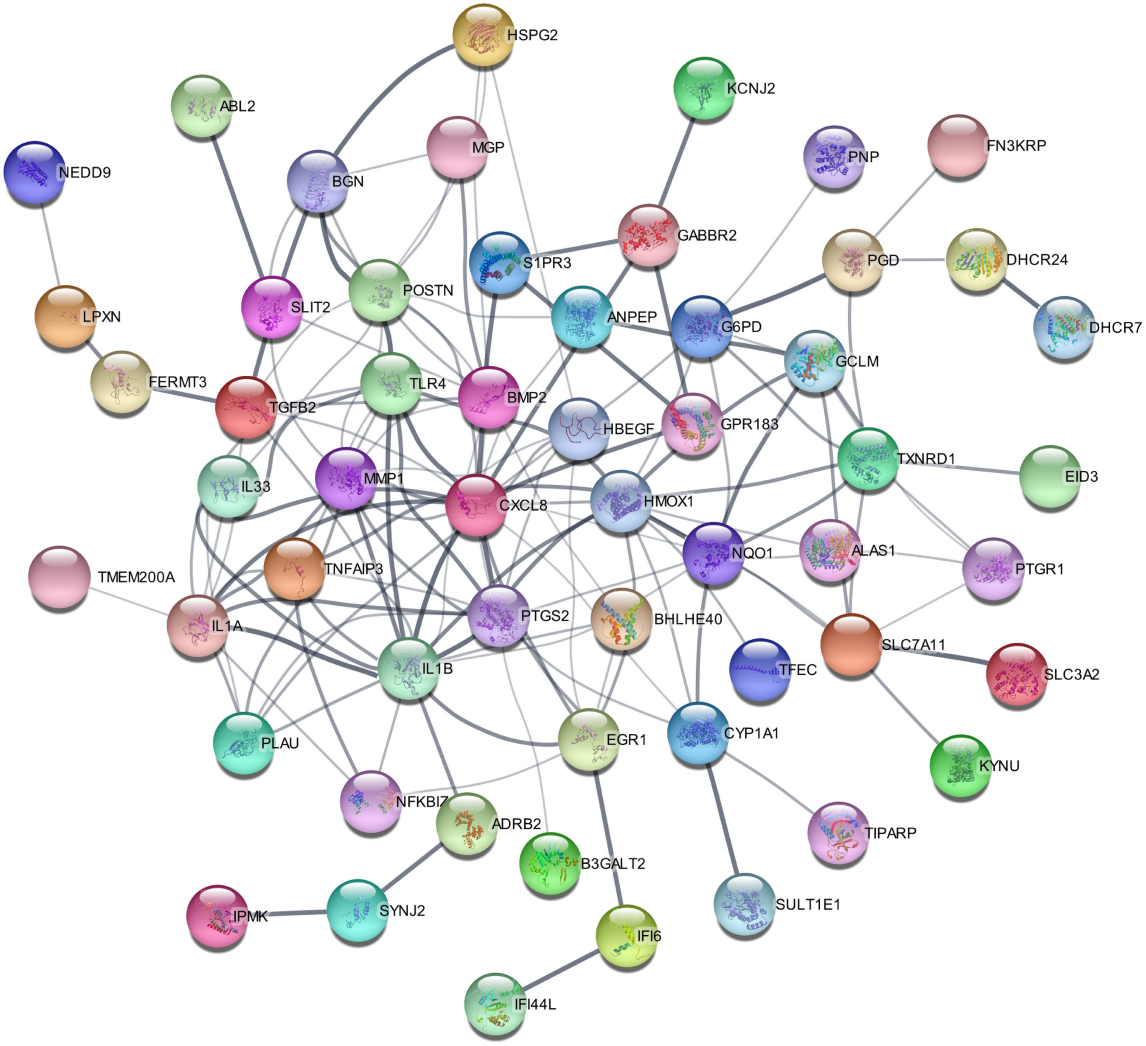
PPI network of 65 DEGs with 140 interaction pairs.

To construct the protein-protein interaction network and identify hub genes in HPMECs exposed to cyclic stretch, STRING database was used to predict the interactions of DEGs, and Cytoscape was used for visualization. For the 97 DEGs, the PPI network contained 65 nodes and 140 edges (Figure 3). The top nine most significantly hub genes were *PTGS2, MMP1, IL1A, TLR4, TNFAIP3, EGR1, HMOXQ, CYP1A1*, and *PLAU*.

### LncRNA-mRNA Coexpression Networks

**Figure 4:**
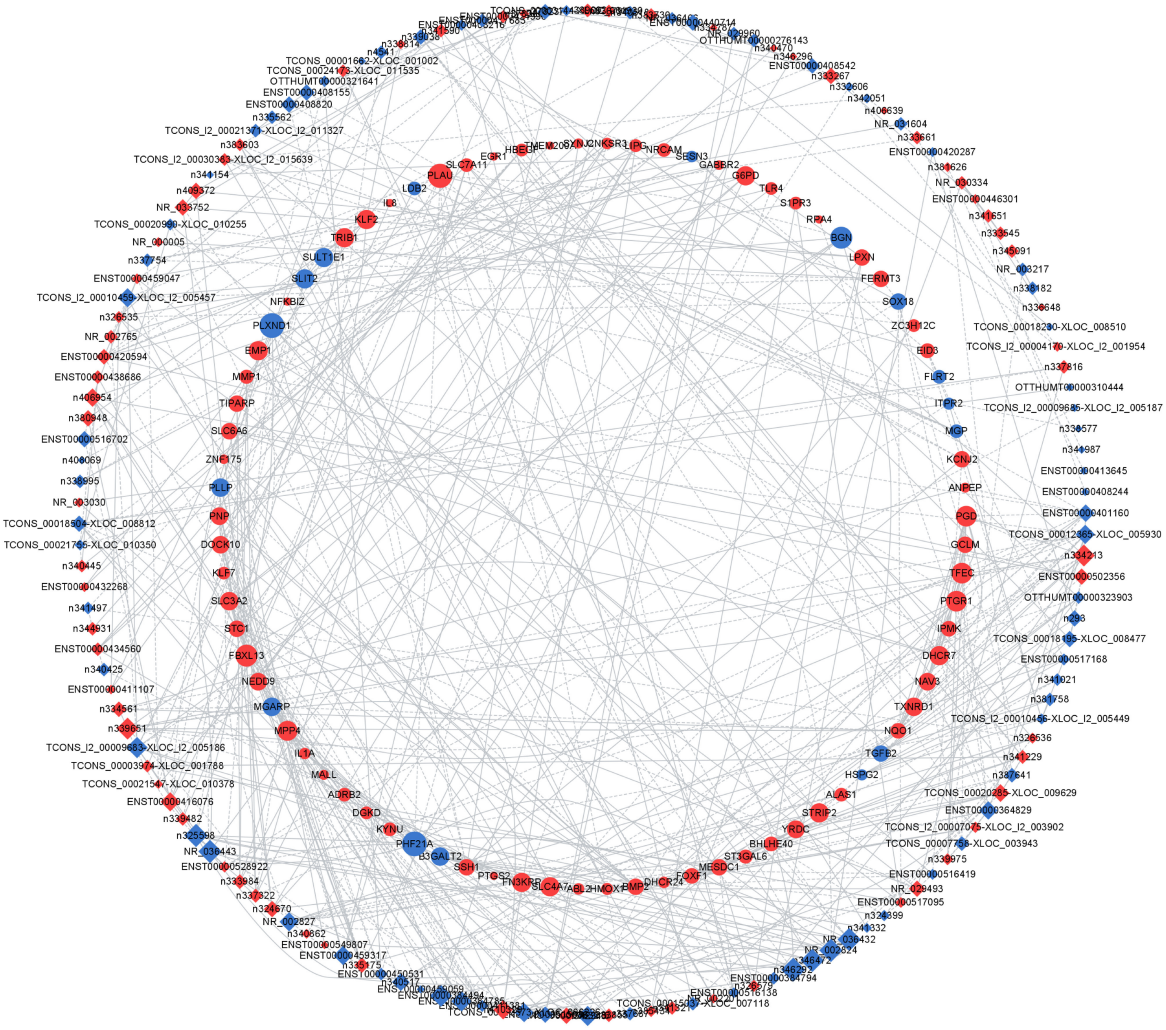
Coexpression network of 149 DELs and 82 interacting DEGs. The diamonds represent lncRNAs; the circles represent their correlated mRNAs. Blue dots and red dots indicate downregulated and upregulated lncRNAs or mRNAs, respectively. Circle size indicates the node degree.

**Figure 5:**
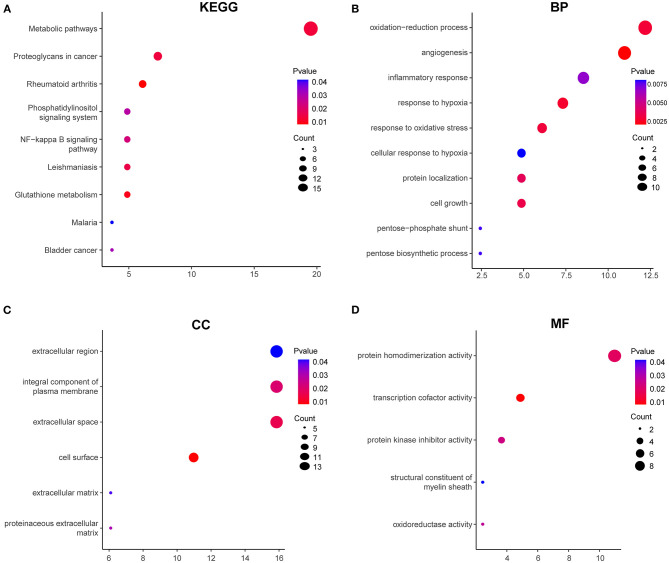
GO functional and KEGG enrichment analyses of 82 DEGs in the coexpression network. **(A)** Significantly enriched KEGG pathways. GO functional enrichment analysis includes BP **(B)**, CC **(C)**, and MF **(D)**. Red to blue colors indicate low to high levels of *p*-value. Point size indicates the number of DEGs in the corresponding pathway.

To address the potential functions of DELs in HPMECs subjected to cyclic stretch, we built a lncRNA-mRNA coexpression network that contained 149 DELs, 82 interacting DEGs, and 140 interaction pairs (Figure 4). KEGG pathway enrichment analysis demonstrated that the Metabolic pathways and NF-kappa B signaling pathway were significantly enriched, as shown in Figure 5A. GO functional enrichment analysis suggested that 82 DEGs in the network were significantly enriched in oxidation-reduction process, angiogenesis, inflammatory response, response to hypoxia, response to oxidative stress, integral component of plasma membrane, extracellular space, cell surface, protein homodimerization activity, and transcription cofactor activity (Figures 5B–D). LncRNA *n333984* and *n337322* were significantly coexpressed with *MMP1* mRNAs in the lncRNA-mRNA coexpression network.

### Cis-Analyses of DELs

To identify the cis-target genes of DELs, we searched coding genes within 10 kb upstream and downstream of these lncRNAs. Cis-analyses showed that 93 DELs were adjacent to or overlapped with 60 genes in sense or antisense direction, 10 of which were differentially expressed: *SESN3, SLC3A2, MGP, TLR4, TNFAIP3, MMP1, G6PD, ITGA11, CNKSR3, and TXNRD1* (Table 1).

**Table 1:** Cis-Analysis of differentially expressed lncRNAs.

**DELs**					**Cis-DEGs**			
**Accession number**	**Database**	***p*-value**	**Fold change**	**Feature**	**Gene symbol**	***p*-value**	**Fold change**	**Feature**
*n381758*	NONCODE	0.00036	−1.830061	down	*SESN3*	0.002252	−1.608661	down
*n410329*	NONCODE	0.00569	1.636504	up	*SLC3A2*	0.013663	1.382277	up
*n335562*	NONCODE	0.006288	−1.667897	down	*MGP*	0.016323	−1.463823	down
*n406639*	NONCODE	0.014825	1.313714	up	*TLR4*	0.035155	1.297185	up
*n335470*	NONCODE	0.016481	1.988396	up	*TNFAIP3*	0.044094	1.525564	up
*n337322*	NONCODE	0.016943	1.897594	up	*MMP1*	0.01301	1.87386	up
*n334213*	NONCODE	0.018357	1.521873	up	*G6PD*	0.015567	1.335835	up
*n336648*	NONCODE	0.024504	1.622163	up	*ITGA11*	0.014963	1.496772	up
*n344931*	NONCODE	0.033057	1.348436	up	*CNKSR3*	0.008027	1.550478	up
*ENST00000549807*	ENSEMBL	0.028716	1.598337	up	*TXNRD1*	0.03917	1.490883	up

## Discussion

As one of the primary supportive therapeutic methods for ARDS, mechanical ventilation could increase pulmonary microvascular permeability and inflammatory response, which further aggravates lung injury and leads to VILI (Moraes et al., 2014; Bates and Smith, 2018). The majority of “omic” studies of VILI have been carried out in lung microvascular endothelial cells or other lung cells (Frye et al., 2005; Woods et al., 2015), or in tissues from preclinical models of VILI (Ma et al., 2005; Wang et al., 2018). To study the effects of mechanical ventilation on endothelial cell function, we stimulated HPMEC with 20% cyclic stretch for 2 h, which was an established model of VILI to generate a mechanical-based lung injury via excessive mechanical stress (Birukov, 2009; Abiko et al., 2015; Meliton et al., 2015; Tian et al., 2016). Previously, the lncRNA expression profile was analyzed using transcriptomics of the “one-hit” mouse model of VILI (Wang et al., 2018). Different from the previous literatures, our study focused on the expression profile and transcriptional functions of endothelium-related lncRNAs associated with VILI. This is the first study to reveal the expression profile and potential roles of lncRNAs in HPMECs subjected to cyclic stretch. We have identified 199 DELs and 97 DEGs in HPMECs subjected to cyclic stretch. Integrated bioinformatics analysis demonstrated that DELs might be involved in response to hypoxia, response to oxidative stress, inflammatory response, pentose biosynthetic process, and cellular response to hypoxia. LncRNA *n335470, n333984, n337322*, and *n406639* may regulate inflammation and fibrosis induced by cyclic stretch through cis- or trans-acting mechanisms. These results provide novel perspectives of the potential molecular mechanisms underlying VILI, as well as implement fundamental evidence for future research.

Long non-coding RNA (lncRNA), whose expression is regulated by various mechanisms, such as chromatin regulation, epigenetic modifications, promoter activity modulation, and post-transcriptional mechanisms, is usually defined as transcripts longer than 200 nucleotides with no protein-coding function (Rinn and Chang, 2012; Batista and Chang, 2013; Quinn and Chang, 2016; Tao et al., 2016). It has been reported that lncRNA could regulate gene expression in cis or trans regulatory manner (Elcheva and Spiegelman, 2020; Rinn and Chang, 2020). For cis acting manner, lncRNA loci exerts function to control the expression of neighboring or overlapping genes. This occurs through a variety of mechanisms including, cis-acting DNA-regulatory elements, the promoter region, or the act of transcription. For trans acting manner, lncRNAs regulate gene expression through binding with proteins, DNA, or other RNA, far away from the site of primary locus of transcription.

In this study, *n335470* and *TNFAIP3* were upregulated in the HPMECs in response to mechanical ventilation, with *n335470* location overlapping the *TNFAIP3* gene on the same chromosome. The same pattern holds for *n406639* and *TLR4*. TNFAIP3, which is a ubiquitin-editing enzyme, could restrict inflammatory responses by negatively regulating NF-κB and MAPK signaling pathways (Yu et al., 2014). It has been reported that TNFAIP3 may stabilize the endothelial barrier by regulating the expression of VE-cadherin in acute lung injury (ALI) (Soni et al., 2018). Previous studies have also indicated that endothelial cells-expressed TLR4 increased vascular permeability and inflammatory responses in the early stages of ALI and/or ARDS (Tauseef et al., 2012; Wu et al., 2017; Peng et al., 2019). Its mechanism of action was likely to be that TLR4 induced the immune response by activating NF-κB signaling pathway via phosphorylating IRAK1 and IRAK2. We suggest that *n335470* and *n406639* may act as cis-acting factors of *TNFAIP3* and *TLR4*, respectively, and play important roles in regulation of inflammatory responses and endothelial barrier.

Our results indicated that *MMP1, n333984*, and *n337322* were significantly differentially expressed in HPMECs stimulated with mechanical ventilation, while *n333984* and *n337322* location overlapped with *MMP1* gene on the same chromosome. Moreover, lncRNA-mRNA coexpression networks analysis results showed that *n333984* and *n337322* were significantly coexpressed with *MMP1* mRNA. MMP-1 played an important role in early pulmonary fibrosis through inducing the degradation of collagen type I and collagen type III (Rosas et al., 2008; Yang et al., 2020). It has been reported that MMP-1 could also decrease ROS production, and contribute to a proliferative, migratory, and anti-apoptotic phenotype, suggesting its therapeutic potential (Herrera et al., 2013). Thus, lncRNA *n333984* and *n337322* might be involved in fibrosis, proliferative, migratory, and anti-apoptotic by regulating the expression of *MMP1* through multiple potential mechanisms.

There were several limitations of this study. First, the *in vitro* model had its limitations in fully reflecting biological conditions in patients, even if this VILI model was used and validated in many studies. Therefore, bias in comparison to what occurs in humans may develop. Second, our findings were mainly based on the integrated bioinformatics analysis of a relatively small sample size. Future studies containing sizable sample may be employed to validate the results both *in vivo* and *in vitro* at both protein and RNA level.

In the present study, we screened 199 DELs and explored the potential functions and underlying mechanisms of these lncRNAs through integrated bioinformatics analysis in HPMECs subjected to cyclic stretch. The DELs were mainly involved in NF-kappa B signaling pathway. LncRNA *n335470, n406639, n333984*, and *n337322* might play important regulatory roles through multiple potential mechanisms. Taken together, this study provides the first transcriptomic landscape of differentially expressed lncRNAs in HPMECs subjected to cyclic stretch, which lays the groundwork for future basic and clinical research of VILI.

## Data Availability Statement

The microarray data in this study has been deposited into a publicly accessible repository (accession: GSE166772).

## Author Contributions

DW and CD performed bioinformatics analysis and wrote the manuscript. XZ, CG, and ML performed the experiments. HL, FY, and HW collected the data. YW reviewed and edited the manuscript. All authors contributed to the manuscript and approved the submitted version.

## Conflict of Interest

The authors declare that the research was conducted in the absence of any commercial or financial relationships that could be construed as a potential conflict of interest.
